# Association between diabetes knowledge and medication adherence among people with type 2 diabetes mellitus of Gokarneshwor municipality: A community-based cross-sectional study

**DOI:** 10.1371/journal.pgph.0005635

**Published:** 2025-12-16

**Authors:** Ashna Parajuli, Paras Kumar Pokharel, Ram Bilakshan Sah, Rajendra Karkee, Prajjwal Pyakurel

**Affiliations:** School of Public Health and Community Medicine, B. P Koirala Institute of Health Sciences, Dharan, Nepal; St John's Medical College, INDIA

## Abstract

Successful management of diabetes requires adequate knowledge of diabetes and changes in lifestyle which is considered a key component of diabetes management. This study explored the association between diabetes knowledge and medication adherence of the people living with type 2 diabetes mellitus living in Gokarneshwor municipality. A community-based cross-sectional study was conducted in the wards of Gokarneshwor municipality. The study included a total of 310 participants and was conducted over a period of 1 year. Diabetes Knowledge was measured using Diabetes Knowledge Questionnaire (DKQ) and Medication Adherence was measured using Morisky’s Medication Adherence Scale (MMAS). Analysis was done using SPSS version 11.5. The study found the mean diabetes knowledge score to be 13.18 ± 3.73. Diabetes knowledge did not show any significant association with medication adherence. More than half of the participants (63.2%) had perfect medication adherence. After adjusting for confounding variables in logistic regression, only employment status emerged as a statistically significant predictor of perfect adherence (AOR = 0.492; p-value = 0.006), with employed patients demonstrating lower adherence compared to unemployed patients. The findings suggested that individuals with limited diabetes knowledge can still exhibit high levels of medication adherence, underscoring the complexity of adherence behaviors. The study did not find significant association between diabetes knowledge and medication adherence. Overall the study indicates that factors beyond diabetes knowledge influence medication adherence among people with type 2 diabetes.

## Introduction

Among the NCDs, diabetes has emerged to be one of the most fatal and prevalent diseases as time progresses. According to WHO in 2021 approximately 537 million adults of 20–79 years of age are suffering from diabetes and it is expected to reach 643 million by the year 2030 and 783 million by 2045. Almost 81% of the adults suffering from diabetes are from low and middle-income countries [1]. In 2021, 6.7 million deaths were caused by diabetes. The burden of deaths due to diabetes has been in increasing trends [1].

In 2024, the prevalence of diabetes among adults in Nepal was approximately 7.7% [2].WHO estimated that the number of diabetes cases in Nepal will rise to about 1,328,000 cases by 2030 [3]. According to non-communicable disease STEPS Survey 2019, only 55% of adults who were prescribed medication for increased blood sugar were actively taking it. Among those receiving treatment, 6% had their blood sugar effectively controlled, while 14.7% still had uncontrolled blood sugar levels. Additionally, 73.5% of adults were not aware of their raised blood sugar status [4].

Research shows that improved glycemic control is correlated with improvement in self-care behavior [5–10], increased knowledge in diabetes [11–13], and greater adherence to medication [14–17]. Patients who have poor knowledge of the disease are hospitalized more often due to uncontrolled diabetes [18]. Thus, patients’ knowledge about diabetes is an essential tool in controlling diabetes mellitus [19]. Studies have shown that the attitude of people towards management of diabetes may be affected by low levels of knowledge of the disease, not being able to understand instructions given by doctors and also the fear of the side effects of taking medications for a long time [20]. Successful management of diabetes requires changes in lifestyle and adequate knowledge of diabetes which is considered a key component of diabetes management [21]. Diabetes health education in Nepal is given only through short counseling only to the diabetes patients in health-care facilities [22]. Little is known about diabetes knowledge and its association with medication adherence in Nepal. This study could contribute to understanding the association between the knowledge of diabetes and medication adherence in people diagnosed with diabetes and their effect on blood glucose status of participants.

### Objectives

- To explore the association between diabetes knowledge and medication adherence among people living with type 2 diabetes mellitus living in Gokarneshwor municipality.
- To assess the socio-demographic profile of diabetic patients and determine its association with medication adherence.

## Materials and methods

### Study design, study period and settings

This is a community-based cross-sectional study. The data for this study was collected from 20^th^ February, 2021–10^th^ April, 2021. The study was conducted in Gokarneshwor municipality in Kathmandu District, in the Bagmati Province of Nepal that was established on 2nd December 2014 by merging the former Village development committees Sundarijal, Nayapati, Baluwa, Jorpati and Gokarna. According to the 2068 B.S National census, the population of this municipality was 1,07,351 with 1768 households (88). Considering the recent increase, it can be estimated that the population is more than one lakh fifty thousand. It is a peri-urban area of Kathmandu which is rapidly urbanizing with soaring in-migration. Peri-urban areas act as the rural and urban transition zone. The area is chosen from the newly established municipality of Kathmandu.

### Study population

The study population were residents of Gokarneshwor municipality residing for more than 6 months.

Inclusion and exclusion criteria:

The eligibility criteria of the participants were (1) People diagnosed (confirmed by participant’s medical records taken from the OPD card or their hospital file that the patients have with them) with type II Diabetes Mellitus who were more than 35 years of age. (2) People who were willing to participate in the study. The exclusion criteria were (1) People with type I Diabetes Mellitus, (2) Pregnant women

### Sampling and sample size determination

The sampling technique used in this study was a multistage approach design. Gokarneshwor municipality from Kathmandu district was taken purposively. Five wards out of nine wards from Gokarneshwor municipality were selected randomly using a lottery method. The randomly selected wards were 4, 5, 7, 8 and 9. Equal number of participants were allocated to each of the five selected ward. Household was selected from each ward using spin the bottle method. After reaching the selected area a bottle was rotated in front of the ward office and the house where the bottle pointed was taken as the starting point and the consecutive houses was taken until the allocated sample size was met for that respective ward, If the allocated sample size could not be met in that direction, the bottle was re-rotated in front of the ward office and consecutive houses in another direction was taken. If more than one participant was present in the house, all the participants meeting the inclusion criteria were selected. With 95% CI, margin of error 6%, good knowledge of diabetes among participants to be 60% [23], after adding 20% non-response rate, the sample size calculated was 307. For dividing the sample equally in 5 wards, the sample size of 310 was taken.

### Data collection methods and tools

Data collection was done by face to face interview using structured questionnaires. Demographic data included age, gender, religion, education, marital status, family type, employment status. Clinical characteristics of the participant included duration of diabetes, family history of diabetes, co-morbidities, multiple medication use. Medication adherence was assessed using Morisky’s Medication Adherence four item scale (MMAS) [24]. Diabetes Knowledge was assessed using Diabetes Knowledge Questionnaire (DKQ) 24 item questionnaire [25]. The score is the total percentage of the total items scored as correct. The higher the score, the greater the knowledge.

### Operational definitions

**Employment status:**

It was categorized as:

**Government employee:** Those working for the government.
**Non-government employee:** Those working for the private companies.
**Self-employed:** Those having their own big or small businesses like the shop, transport, hotels etc.
**Non-paid:** Those working on a volunteer basis without any form of wage in return.
**Student:** Those studying and dependent on others for income to run daily lives.
**Homemaker:** Those involved in non-economic activities in home like cleaning, cooking, looking after the cattle.
**Retired:** Those that receive pension.
**Unemployed (unable to work):** Those who were elderly and couldn’t work or were disabled.
**Others:** Those that couldn’t be categorized on any of the above-mentioned employment statuses.

### Marital status

It is classified as per the central bureau of statistics, 2016 as per the Government of Nepal. Single, married, multiple marriage and remarried were merged into the married category.

### Education status

It was classified according to the Education system of Nepal (CBS 2011) Illiterate referred to not being able to read or write. Informal education referred to not attending any formal education in school. Less than Primary referred to studied less than class 5. Primary education referred to completed education up to class 5. Lower secondary referred to completed education up to class 8. Secondary referred to completed education up to 10. Higher Secondary Education referred to completed education up to class 12. Graduate referred to completed bachelor’s degree. Postgraduate referred to completed master’s degree.

### Diabetes knowledge

Knowledge in this study was defined as an understanding of facts, information regarding diabetes which was assessed with diabetes knowledge questionnaire (DKQ) which elicits information about participant’s understanding of diabetes management, management of hypoglycemia, complications, etc [26]. The questionnaire has 24 questions. The mean score received by the participants was calculated for analysis.

### Medication adherence

Medication adherence in this study was measured by Morisky’s medication adherence scale (MMAS) and perfect adherence was defined by scoring aggregate of 4 points by the participants. Participants who were not taking medication at the time of data collection or score less than 4 was defined as non-adherence [24].

### Co-morbidity

Co-morbidity was defined as having one or more than one health problems/disease other than diabetes mellitus. Diagnosis was based on hospital records (OPD cards) shown by the participants.

### Ethical clearance

Ethical clearance was obtained from Institutional Review Committee, BPKIHS, Dharan (**Ref No. 448/077/078 & Code No. IRC/1879/020)**. The purpose of the study was explained to the participants and informed consent was taken before commencing the interview.

### Data analysis

Frequency, percentage, mean, standard deviation, median and interquartile range were used to describe and summarize the data and graphical and tabular presentation was made (bar diagram, Pie-charts, etc.). Independent t-test was done to find the difference in diabetes knowledge scores between medication adherent and non adherent groups. Chi-square test was done to find the association between Medication adherence and socio-demographic variables.

Univariate and Multivariable logistic regression analysis was carried out to find the significant predictors of diabetes medication adherence. Statistical significance was tested with 95% confidence interval and a p-value less than 0.05 was considered statistically significant.

## Results

### Socio-demographic characteristics of the participants

A total of 310 participants over 35 years of age were included in the study. The mean age of the participants was 56.10 ± 10.06. A maximum number of the participants (31.9%) was between 55–64 years of age and 24.9% (77) of the participants were 64 years and above. Out of the 310 participants, 48.7% of the participants were male whereas 51.3% of the participants were female. Among the participants, majority were Hindu (88.1%). The percentage of married people was 91.6%. Almost equal percentages (49.4% and 50.6%) of the participants were from nuclear and joint families respectively. Among the participants interviewed, 34.5% were illiterate or had informal education and the remaining had formal education with 22.9% having completed secondary school and 4.2% having completed higher education. Regarding employment, 31.0% were homemakers followed by 29.4% self-employed which meant they were involved in big and small business whereas 13.5% were unemployed and unable to work (Table 1).

**Table 1:** Socio-demographic characteristics of the participants.

Characteristics		Frequency	Percentage (%)
Age (in years) (n = 310)	35-44	44	14.2
	45-54	90	29.0
	55-64	99	31.9
	65-74	69	22.3
	75-84	8	2.6
	Mean Age in years ± SD: 56.10 ± 10.061		
Gender (n = 310)	Male	151	48.7
	Female	159	51.3
Religion (n=310)	Hindu	273	88.1
	Buddhism	26	8.4
	Muslim	3	1.0
	Christian	8	2.6
Marital Status (n = 310)	Married	284	91.6
	Widowed	19	6.1
	Married/Separated	5	1.6
	Divorced	1	0.3
	Never Married	1	0.3
Family type (n = 310)	Nuclear family	153	49.4
	Joint family	157	50.6
Education status (n = 310)	Illiterate	107	34.5
Level of education (if literate)	Less than primary school (<class 5)	33	10.6
	Primary School completed (up to class 5)	25	8.1
	Secondary school completed	71	22.9
	Higher secondary school completed	30	9.7
	Bachelor’s degree completed	31	10.0
	Postgraduate completed	13	4.2
Employment status (n = 310)	Government Employee	15	4.8
	Non-government Employee	23	7.4
	Self-employed	91	29.4
	Homemaker	96	31.0
	Retired	31	10.0
	Unemployed (able to work)	10	3.2
	Unemployed (unable to work)	42	13.5
	Others	2	0.7

### Clinical characteristics of the participants

Regarding the duration since diabetes was diagnosed, 41.6% of the participants were diagnosed between 1 and 5 years ago, followed by 26.1% who were diagnosed 6 and 10 years ago, and 15.5% were diagnosed more than 16 years ago. More than half (57.1%) of the participants mentioned that they had a positive family history of diabetes. When asked about associated diseases apart from Diabetes, 55.8% had other diseases and 44.2% had only diabetes. Among the participants, 52.3% of the participants were taking more than one anti-diabetic medication as prescribed (Table 2).

**Table 2:** Clinical characteristics of the participants.

Characteristics		Frequency	Percentage (%)
Duration of Diabetes (n = 310)	1-5	129	41.6
	6-10	81	26.1
	11-15	52	16.8
	>=16	48	15.5
Family history of diabetes (n = 310)	Yes	177	57.1
	No	133	42.9
Co-morbidities (n = 310)	Yes	173	55.8
	No	137	44.2
Multiple antidiabetic medication use (n = 310)	Yes	162	52.3
	No	148	47.7

**Fig 1:**
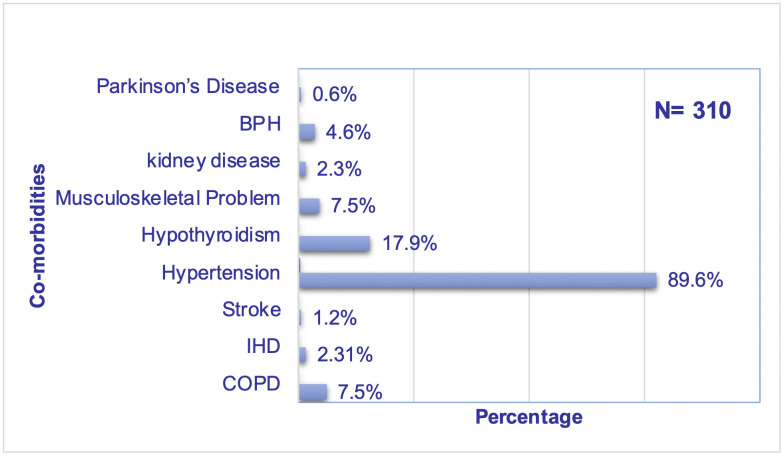
Co-morbidities among the respondents.

Among all participants, who had associated morbidities along with diabetes, 89.6% had hypertension, 17.9% had hypothyroidism, 7.5% had chronic airway disease, 7.5% had musculoskeletal problems, 2.3% had ischemic heart disease and the remaining 8.7% had other diseases (Fig 1).

### Diabetes knowledge

The mean ± SD DKQ score (out of a possible 24) of the respondents was 13.18 ± 3.731. Maximum number of participants (89.0%) believed that diabetes could damage their kidneys. Around 88.7% believed that regular exercise will not increase the need for insulin or other diabetic medication. Likewise, 84.2% participants revealed that diabetics should take extra care when cutting their toenails. More than two-thirds of the participants were well acquainted that diabetes is incurable, causes loss of feelings in hands, fingers, and feet is hereditary. About 80.0% of the participants knew that blood sugar level of 210 is high. Furthermore, less than one-third of the patients had knowledge about types of diabetes, the role of insulin in diabetes, insulin reaction, care of the wound, and the importance of tight elastic hoses or socks (Table 3).

**Table 3:** Diabetes knowledge Questionnaire (DKQ-24) results of the participants.

Diabetes knowledge Questions	Frequency*	Percentage
Eating too much sugar and other sweet foods is a cause of diabetes	129	41.6%
The usual cause of diabetes is lack of effective insulin in the body	99	31.9%
Diabetes is caused by failure of the kidneys to keep sugar out of the urine	32	10.3%
Kidneys produce insulin	56	18.1%
In untreated diabetes, the amount of sugar in the blood usually increases	197	63.5%
If I am diabetic, my children have a higher chance of being diabetic	208	67.1%
Diabetes can be cured	212	68.4%
A fasting blood sugar level of 210 is too high	248	80.0%
The best way to check my diabetes is by testing my urine	219	70.6%
Regular exercise will increase the need for insulin or other diabetic medication	275	88.7%
There are two main types of diabetes: type 1 (insulin-dependent) and type 2 (non-insulin dependent)	78	25.2%
An insulin reaction is caused by too much food	52	16.8%
Medication is more important than diet and exercise to control my diabetes	223	71.9%
Diabetes often causes poor circulation	121	39.0%
Cuts and abrasions on diabetics heal more slowly	256	82.6%
Diabetics should take extra care when cutting their toenails	261	84.2%
A person with diabetes should cleanse a cut with iodine and alcohol	61	19.7%
The way I prepare my food is as important as the foods I eat	241	77.7%
Diabetes can damage my kidneys	276	89.0%
Diabetes can cause loss of feeling in my hands, fingers, and feet	246	79.4%
Shaking and sweating are signs of high blood sugar	111	35.8%
Frequent urination and thirst are signs of low blood sugar	224	72.3%
Tight elastic hose or socks are not bad for diabetics	137	44.2%
A diabetic diet consists mostly of special foods	125	40.3%

(* = Answered correctly).

### Medication adherence

**Fig 2:**
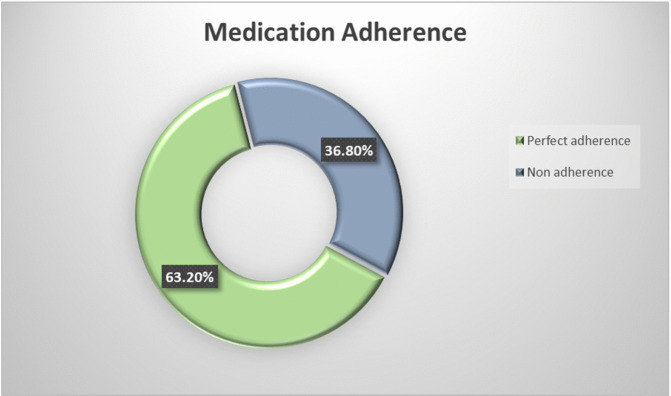
Medication Adherence of the participants.

Perfect adherence was seen in 63.2% of the cases according to the MMAS-4 scale (Fig 2). Among the four reasons for non-adherence assessed by MMAS-4, forgetfulness (74.6%) was the most common cause among those who were non-adherent.

### Association of diabetes knowledge with medication adherence

Respondents who do not have perfect adherence scored more in diabetes knowledge than those with perfect adherence. The observed difference in diabetes knowledge between two adherence groups is not statistically significant (Table 4).

**Table 4:** Association of Diabetes knowledge with Medication adherence.

Characteristics		DKQ Score (mean ± SD)	P-value
Medication adherence (n = 310)	Perfect adherence	12.90 ± 3.714	0.082
	Non-adherence	13.67 ± 3.729	

### Medication adherence

#### Association of different socio-demographic characteristics with adherence.

Mediation adherence was seen higher in females compared to males (p = 0.026). Hindu participants were found to be more adherent to diabetes medication compared to non-Hindu and this was statistically significant (p = 0.002). There was a statistically significant association (p = 0.021) between adherence and education. Adherence was higher in participants who were illiterate than literate. There was higher adherence in participants who were not working compared to participants who were currently working (p = 0.001). Participants in the age group of 35–59 had shown higher adherence than the participants of 60years and above age group.

No Statistical significance was observed in the context of age, marital status, family type and family history of diabetes (Table 5).

**Table 5:** Association of Medication adherence with Socio-demographic variables.

Characteristics		Adherence		Total	P-value
		Perfect adherence	Non-adherence		
Age (n = 310)	35-59 years	112(61.2)	71(38.8)	183(100)	0.375
	60 years and above	84(66.14)	43(33.86)	127(100)	
Gender (n = 310)	Male	86(56.95)	65(43.05)	151(100)	**0.026**
	Female	110(69.2)	49(30.8)	159(100)	
Religion (n = 310)	Hindu	181(66.30)	92(33.7)	273(100)	**0.002**
	non-Hindu	15(40.54)	22(59.46)	37(100)	
Marital status (n = 310)	Married	178(62.68)	106(37.32)	284(100)	0.507
	Others	18(69.23)	8(30.77)	26(100)	
Education status (n = 310)	Literate	119(58.62)	84(41.38)	203(100)	**0.021**
	Illiterate	77(71.96)	30(28.04)	107(100)	
Employment status (n = 310)	Currently working	68(52.71)	61(47.29)	129(100)	**0.001**
	Currently not working	128(71.51)	51(28.49)	179(100)	
Family Type (n = 310)	Nuclear family	95(62.09)	58(37.91)	153(100)	0.683
	Joint family	101(64.33)	56(35.67)	157(100)	
Family history of Diabetes (n = 310)	Yes	108(61.0)	69(39.0)	177(100)	**0.035**
	No	88(66.2)	45(33.8)	133(100)	

#### Association of clinical characteristics with medication adherence.

Medication adherence was significantly associated with family history of diabetes. In terms of duration of illness, participants with 1–10 years of diabetes were more non-adherent compared to participants diagnosed for more than 10 years but this was not statistically significant. Adherence was higher in patients taking multiple pills compared to a single medication. Higher adherence was seen in participants suffering from co-morbidity compared to those suffering from diabetes only. This association was, however, not statistically significant.

### Multivariable analysis

All the variables which were associated at 20% significance (p ≤ 0.2) in univariate analysis were included into multivariable logistic regression analysis. Analysis was done after the significantly associated variables were checked for the multi-collinearity. No collinearity was found among the variables.

Participants who were currently working were less likely (AOR = 0.492; CI = 0.297-0.815) to be adherent to anti-diabetic medication compared to those who were not working currently and this finding was statistically significant (Table 6).

**Table 6:** Multiple variable analysis of factors associated with medication adherence.

Characteristics		β Coefficient	Adjusted Odds Ratio	p-value	95% CI	
					Lower	Upper
Education status	Literate	-0.310	0.733	0.264	0.425	1.264
	Illiterate	Ref				
Employment Status	Currently working	-0.709	0.492	**0.006**	0.297	0.815
	Currently not working	Ref				

## Discussion

### Diabetes knowledge

Findings from this study revealed that diabetes knowledge was inadequate among the respondents with a mean score of 13.18 ± 3.73 which was consistent with the study done in Kerala with a mean score of 13.3 ± 2.6 [27].

Our study showed no significant association between diabetes knowledge and medication adherence. A study in Pakistan showed a significant but weak positive correlation between diabetes-related knowledge and medication adherence (r = 0.036, P < .05) [28]. Even though participants have adequate diabetes–related knowledge, adherence to treatment was poor among the participants [28]. Likewise, a study done in Malaysia found that having good or poor knowledge of diabetes does not guarantee health seeking practice of medication adherence [29]. In contrast to the finding, a study done in Palestine showed significant association of diabetes knowledge with medication adherence. Those who showed high diabetes-related knowledge score were less likely to be non-adherent [O.R = 0.8; 95% C.I of 0.7 - 0.9] [30]. The contrast finding in our study might be due to the use of different adherence scale in our study compared with other studies that used MMAS-8 to assess the medication adherence. Also, our study was conducted in a community setting where the probability of finding participants with good diabetes knowledge scores and adherence pattern may be low as compared to the findings from the study done in hospital setting.

### Medication adherence

In this study, perfect adherence was seen among 63.2% of the participants which is similar to the finding of a study by Mutyambizi et al. where medication adherence was found to be among 67% of the participants [31]. Our study shows higher adherence compared to the findings of Sah BK et al. who reported 65.1% of the participants in a tertiary hospital were non-adherent [32]. A study done in Letang, Nepal showed about 54.1% of the participants were found to be adherent to medication [33]. Increasing awareness about diabetes mellitus and its complications over the years and increase in attention towards management of diabetes might be one of the explanations of higher adherence to medication in this study. Forgetfulness was the most common reason given by 74.6% of the non-adherent responders in this study. This result was similar to the results of another study by Aminde et al. which highlighted that forgetfulness was one of the main reasons for non-adherence [34].

### Medication adherence and associated factors

The results of this study revealed that females were more adherent to diabetes medication compared to males and this difference was statistically significant (p = 0.026). The finding was similar to a study done in Cambodia where a higher level of diabetes medication adherence was significantly higher in females (53.7%, p = 0.004) [35]. One of the reasons for female to have more adherence to medication in our study might also be explained by the fact that those respondents who were currently working had lower medication adherence and, in this study, higher number of males were working, and females were homemakers. However, there was no statistically significant association between gender and medication adherence in multivariable logistic regression analysis in our study which was consistent with the study done in a refugee camp in Beirut, Lebanon [36].

[36] Our study did not show any significant association between education status and medication adherence in logistic regression analysis. In contrast to the result, a study done in hospitals of Kathmandu showed that illiterate participants were 4.32 times more likely to be non-adherent to diabetes medication than literate (p = 0.001) [37]. This difference in our study could be due to the use of different questionnaire and methodologies in other studies.

Participants who were not currently working were found to be more adherent to medication and this was found to be statistically significant in our study (p = 0.001). A study in Dhaka found that employed participants were significantly non-adherent to diabetes medication [38]. This finding of our study was statistically significant in a multivariable logistic regression analysis where those who were working currently were less likely (AOR = 0.49) to be adherent to medication. This result was similar to another study by Sah BK et al. where the working group was 8.2 times more likely to fail to take their medication compared to the non-working group [32]. Being professionally active was significantly associated with poor adherence to medication was reported by Tiv et al. (OR = 1.5) [39]. The reason for this could be those who are working forget or are late to take their medication due to their busy schedule and stressful jobs. Other employment related factors influencing adherence could be further explored in future studies.

## Conclusion

The mean score of diabetes knowledge of the participants was found to be 13.18 ± 3.73. Adherence to diabetes medication was found in a maximum number of respondents. However, there was no significant association between diabetes knowledge and medication adherence of the respondents. Females were more adherent to diabetes medication. Employment status was found to be a significant predictor of perfect medication adherence.

## Limitations

1) Being a self-reported data, over-estimation or under-estimation of medication adherence might have occurred, which may have affected the findings of the study.
2) Social desirability bias could have affected the results. It is reasonable that some patients may have biased answers while trying to follow the more socially desirable options rather than their actual behavior while answering the questions of medication adherence and on the information regarding smoking and alcohol intake.
3) In some cases, recall bias could not be minimized with regard to duration of diagnosis of disease due to limited information on the prescription.
4) Our study did not differentiate the participants with or without diabetes related complications. Even though the study probably included such people, the absence of data on the type or severity of complications may have affected our ability to explore their potential impact on knowledge and adherence.
5) Cross-sectional nature of the study limits its ability to draw causal inferences.

## Supporting information

S1 Data Dataset. (XLSX)
